# Inflammatory Myopathy: When Electromyography and Autoantibodies Don’t Help the Diagnosis

**DOI:** 10.7759/cureus.53569

**Published:** 2024-02-04

**Authors:** Anabela De Carvalho, Luís Filipe Couto, Filipe Gonçalves, Jorge Cotter

**Affiliations:** 1 Internal Medicine, Unidade Local de Saúde do Alto Ave, Guimarães, PRT

**Keywords:** corticosteroids, myositis-specific autoantibodies, subacute-onset muscle weakness, idiopathic inflammatory myopathies, autoimmune necrotizing myositis

## Abstract

Inflammatory myopathies (IM) are the most treatable myopathies. Necrotizing autoimmune myositis is a distinct clinicopathologic entity that starts either acutely or subacutely. Autoimmunity is essencial in the pathogenesis of myositis and autoantibodies may be present in more than 50% of patients. We present the case of a 73-year-old man with elevated levels of CK and aldolase, and proximal symmetric muscle weakness and weight loss. The etiological investigation revealed, via muscle biopsy, a necrotizing autoimmune myositis, even though the majority of usual autoantibodies and electromyography were negative. The case demonstrates the importance of combining the patient’s symptoms, neurological examination, and analytical changes to corroborate the suspicion of myopathy.

## Introduction

Inflammatory myopathies (IM) are the largest group of potentially treatable myopathies in children and adults [1,2]. They may be classified into four subtypes: dermatomyositis, polymyositis, necrotizing autoimmune myositis, and inclusion-body myositis [1]. Due to an abnormal reaction of the immune system, striated muscles become gradually inflamed, and the number of rhabdomyocytes responding to each signal given by motor units reduces, leading to symmetric muscle weakness [3].

Necrotizing autoimmune myositis accounts for up to 19% of all inflammatory myopathies [1,4]. It can occur at any age but is seen primarily in adults. Patients usually present with severe proximal weakness of acute or subacute onset, and show very high creatine kinase (CK) levels. This myositis occurs alone or after viral infections, in association with cancer, in patients with connective-tissue disorders such as scleroderma, or in patients taking statins. Most patients have antibodies against signal recognition particle (SRP) or against 3-hydroxy-3-methylglutaryl-coenzyme A reductase (HMGCR). The initial treatment option is prednisone (1 mg per kilogram) for 4 to 6 weeks with posterior dose tapering. Glucocorticoid-sparing agents such as azathioprine, methotrexate, and mycophenolate may be used. If the first line of treatment fails, intravenous immunoglobulin over a period of 2 to 5 consecutive days is used [1].

This article describes a case of a 73-year-old man who went to the Emergency Department (ED) with elevated levels of CK (4168 IU/L); normal range 46-171 IU/L] and aldolase (65.5 IU/L; normal range <7.6 IU/L), with proximal symmetric muscle weakness and weight loss. The etiological study showed necrotizing autoimmune myositis, despite most typical autoantibodies being negative using muscle biopsy, demonstrating the importance of clinical physical exam and muscle biopsy findings in the classification of a myopathy.

## Case presentation

A 73-year-old independent man with a history of dyslipidemia, transient ischemic attack, peptic ulcer disease, and previous smoking habits was referred to the ED due to analytical changes. The patient presented bilaterally decreased muscle strength in the upper and lower limbs, associated with myalgia, asthenia, and weight loss of 12% of body mass over 7 months. He brought an analytical study with leukocytosis and elevated creatine kinase (CK) and aldolase. The patient was chronically treated with statins for more than 10 years. The patient denied dysphagia, gastroesophageal reflux, skin changes, arthralgias, respiratory complaints, trauma, or changes in his usual medication. The neurological examination revealed predominantly proximal symmetric tetraparesis (muscular strength (MS) of the shoulders grade (G) 2/5, elbow G4/5, hips G2/5, and knees G4/5, without changes in the wrist, hands, ankles, and feet) associated with decreased muscle strength in neck flexion. Muscle tone and osteotendinous reflexes were normal. A repeat analytic study showed liver cytolysis, elevated lactic dehydrogenase, CK, and myoglobin (Table 1).

**Table 1:** Laboratory findings on admission to the ED IU/L: International Units per Liter

Laboratory test	Result	Normal range
Aspartate aminotransferase	232 IU/L	12-40 IU/L
Alanine aminotransferase	316 IU/L	7-40 IU/L
Lactic dehydrogenase	587 IU/L	120-246 IU/L
Creatine kinase	5159 IU/L	46-171 IU/L
Myoglobin	2689 IU/L	14-106 IU/L

A thoracoabdominopelvic computed tomography was performed which documented a bilateral paraseptal and centrilobular emphysema and apparent thickening of the walls of the gastric antrum, with slightly irregular contours, but without other changes. The patient was hospitalized due to suspected myopathy for study in the Internal Medicine department, with suspension of statin therapy.

The autoimmune study was positive for anti-nuclear antibodies (ANA) 1/160, anti-Ku, and anti-PMScl100, with a negative result for anti-SRP and anti-HMGCR. Electromyography of the limbs, echocardiogram, and functional respiratory tests were unremarkable. A muscle biopsy was performed on the right deltoid which revealed the presence of atrophied fibers with rounded contours dispersed throughout the fascicles. Necrotic fibers were frequently identified, sometimes undergoing phagocytosis, isolated and not associated with inflammatory infiltrates. There were even rarer atrophied fibers with a basophilic appearance and at least one viable fiber being invaded by macrophages. Major histocompatibility complex (MHC) class I antigen with diffuse sarcolemic and sarcoplasmic positivity observed in some fibers. These were suspected to be due to immune-mediated necrotizing inflammatory myopathy. 

The paraneoplastic study carried out documented a previously known thyroid nodule without evolution, upper digestive endoscopy with non-erosive gastropathy, and colonoscopy with colonic diverticulosis.

The patient was started on corticosteroids at 1 mg/kg/ day. After 11 days of therapy, he presented with improvement in MS as follows: shoulders G3-/5, elbows G4+/5, hips G2+/5, and knees G4+/5. He began an inpatient rehabilitation program, continued in an outpatient setting, and was referred for external Internal Medicine consultation.

At reassessment 6 weeks after discharge, the patient showed a continued improvement in MS: shoulders G4/5, elbows G4+/5, hips G3/5, and knees G4+/5. Improvement was also seen analytically (Table 2).

**Table 2:** Comparison of analytical changes since hospital admission and six weeks after discharge ED: Emergency Department; IU/L: International Units per Liter

Laboratory test	ED result	Consultation result	Normal range
Aspartate aminotransferase	232 IU/L	54 IU/L	12-40 IU/L
Alanine aminotransferase	316 IU/L	129 IU/L	7-40 IU/L
Lactic dehydrogenase	587 IU/L	262 IU/L	120-246 IU/L
Creatine kinase	5159 IU/L	401 IU/L	46-171 IU/L
Myoglobin	2689 IU/L	302 IU/L	14-106 IU/L

The patient began to reduce corticosteroids, by decreasing 5mg every 2 weeks until 20mg, and started methotrexate at 10mg per week.

## Discussion

The diagnosis of the exact subtype of inflammatory myopathy is based on the combination of clinical history, disease progression, pattern of muscle involvement, muscle enzyme levels, electromyographic findings, muscle-biopsy analysis, and for some conditions, the presence of certain autoantibodies [1]. Differential diagnosis includes drug toxicity, viral infection, endocrinological causes (hypo/hyperthyroidism and potassium disturbances), myasthenia gravis, paraneoplastic syndrome, vasculitis, and infiltrative myopathy [3,5].

Electromyography is used to rule out neurogenic conditions and assess disease activity, while muscle biopsy is essential for the diagnosis of polymyositis, overlap myositis, necrotizing autoimmune myositis, and inclusion-body myositis, helping to rule out dystrophies or metabolic myopathies [1].

Autoimmunity is believed to have a key role in the pathogenesis of myositis and autoantibodies have been identified in over 50% of patients with IM. Myositis-specific autoantibodies and myositis-associated autoantibodies have been extensively demonstrated to correlate with specific clinical manifestations and are important biomarkers for myositis, aiding in diagnosis and helping to classify patients into more homogeneous groups [2].

The case presented reinforces the importance of combining the patient’s symptoms, neurological examination, and analytical changes to corroborate the suspicion of myopathy, and to continue the etiological study with muscle biopsy, despite negative electromyography and autoantibodies.

## Conclusions

The case presented is interesting not only because of the slow progression but also due to negative electromyography and the absence of typical autoantibodies, which may lead to the non-realization of muscle biopsy. Due to the suspicion of myopathy, the diagnosis of necrotizing autoimmune myositis was made and the patient initiated adequate treatment, with documented MS improvement within weeks. This case reinforces the importance of muscle biopsy as the gold standard for diagnosis. 
